# Early Postoperative Circulating miR-483-5p Is a Prognosis Marker for Adrenocortical Cancer

**DOI:** 10.3390/cancers12030724

**Published:** 2020-03-19

**Authors:** Maurine Oreglia, Silviu Sbiera, Martin Fassnacht, Laurent Guyon, Josiane Denis, Justine Cristante, Olivier Chabre, Nadia Cherradi

**Affiliations:** 1 Centre Hospitalier Universitaire Grenoble Alpes, Service d’Endocrinologie, F-38000 Grenoble, France; maurine.oreglia@ch-metropole-savoie.fr (M.O.); jcristante@chu-grenoble.fr (J.C.); OlivierChabre@chu-grenoble.fr (O.C.); 2 Department of Internal Medicine I, Endocrinology and Diabetes Unit, University Hospital Würzburg, 97080 Würzburg, Germany; Sbiera_S@ukw.de (S.S.); Fassnacht_M@ukw.de (M.F.); 3 Univ. Grenoble Alpes, INSERM, CEA, IRIG, Biology of Cancer and Infection UMR_S 1036, F-38000 Grenoble, France; laurent.guyon@cea.fr (L.G.); josiane.denis@cea.fr (J.D.)

**Keywords:** adrenocortical carcinoma, biomarker, circulating microRNA, miR-483-5p, early prognosis, recurrence

## Abstract

We have previously identified serum miR-483-5p as a preoperative diagnosis and prognosis biomarker for adrenocortical cancer (ACC). Here, we aimed to determine whether circulating miR-483-5p levels measured 3 months post-operatively distinguished patients with good prognosis (no recurrence for at least 3 years; NR3yrs) from patients with poor prognosis (recurrence or death within 3 years after surgery; R < 3yrs). We conducted a single-center retrospective analysis using sera from 48 patients with ACC that were initially non-metastatic and treated by surgery. Sera sampled within 3 months after surgery were available in 26 patients. MiR-483-5p absolute circulating levels were measured using quantitative PCR. Thirteen patients showed a recurrence before 3 years (=R < 3yrs). Thirteen patients showed no recurrence within 3 years, including 11 patients with a follow-up longer than 3 years (=NR3yrs). Serum miR-483-5p levels were higher in R < 3yrs than in NR3yrs: 1,541,990 ± 428,377 copies/mL vs. 388,457 ± 62,169 copies/mL (*p* = 0.002). Receiver operating characteristic analysis showed that a value of 752,898 copies/mL distinguished R < 3yrs from NR3yrs with 61.5% sensitivity (CI 31.6–86.1) and 100% specificity (CI 71.5–100) with an area under the curve of 0.853. Patients with a value below this threshold had a significantly longer recurrence-free and overall survival. In multivariate analysis, miR-483-5p provided the single best prognostic value for recurrence-free survival (RFS) (hazard ratio (HR) for recurrence 5.98, *p* < 0.011) but not for overall survival. Our study suggests that serum miR-483-5p is a potent early post-operative biomarker for ACC prognosis that might be a better predictor of RFS than currently used markers.

## 1. Introduction

Adrenocortical carcinoma (ACC) is a rare tumor entity with an incidence of 0.7 to 2 per million per year. The prognosis is generally poor with five-year survival not exceeding 40% in most series [1,2,3]. Within this overall estimate, the ENSAT (European Network for the Study of Adrenal Tumors) recommends distribution of patients in four stages, with a 5-year survival rate of 73.9%, 63.8%, 44.1%, and 6.9% for stages I, II, III and IV respectively [1,4]. However, except for patients with distant metastasis at diagnosis, this staging system is not based on parameters directly linked to the persistence of neoplastic cells after surgery, which is likely a major factor for recurrence and thus indirectly for the survival of the patients. In this study, we aimed to define whether circulating microRNA hsa-miR-483-5p, which is likely of tumor origin [5], might provide a better prognosis assessment. MicroRNAs (miRNA) are small 20–22 nucleotides of non-coding RNA which act as post-transcriptional regulators of messenger RNA (mRNA) stability and/or translation [6]. Subsets of miRNAs were shown to be differently expressed between adrenocortical adenomas (ACA) and ACC [7]. MiR-483-5p levels were found to be higher in ACC tumor tissues [5,8,9,10,11] as well as in serum or plasma of patients with ACC as compared to patients with ACA [5,12,13,14]. We first demonstrated the diagnostic and prognostic values of circulating miR-483-5p by showing that serum miR-483-5p measured pre-operatively was significantly higher in patients with aggressive ACC (relapse or death in the first three years post-surgery) than in patients with non-aggressive ACC (at least three years relapse-free survival) [5]. More recently, Salvianti et al. examined post-operative plasma levels of miR-483-5p in a retrospective cohort of 27 ACC patients [14]. In this study, 44 available post-operative samples of 27 patients were pooled together, regardless of the time after surgery. This indicates that some patients were arbitrarily weighted more than others and that the timing of post-operative measurements was not considered. In the present work, we aimed to define more accurately the prognosis value of post-operative serum miR-483-5p in a new cohort of ACC patients by selecting for analysis for each patient the first blood sample collected within three months post-operatively.

## 2. Results

### 2.1. Patients

**Figure 1:**
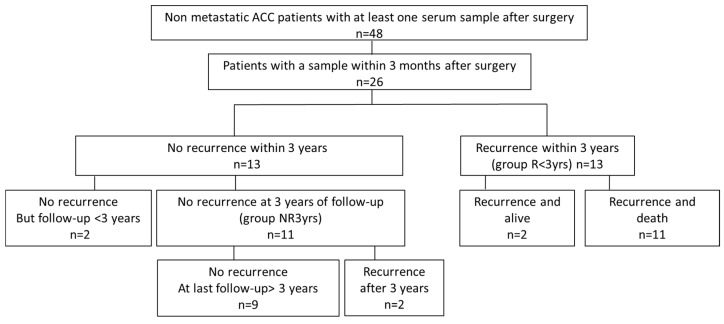
Schematic diagram of the study population. Details are described in the text (“Patients” section). R < 3yrs: patients with recurrence occurring before 3 years post-surgery; NR3yrs: patients with no recurrence within 3 years post-surgery.

Patient clinical characteristics are described in Table 1. Their distribution according to follow-up and recurrence is shown in Figure 1. The 26 patients with blood sampled within 3 months post-operatively included 13 patients who showed a recurrence within 3 years (group R < 3yrs) and two patients who showed a late recurrence, which occurred at 4.75 and 4.2 years (57 and 50 months), respectively. By contrast, 13 patients showed no recurrence during the first 3 years of follow-up, of which 11 patients were effectively followed for at least 3 years (group NR3yrs), while two patients showed no recurrence but had a follow-up of less than 3 years. Two of the 11 patients with no recurrence during the first three years of follow-up were the patients (#12 and #13) who showed a recurrence at 4.75 and 4.2 years as already mentioned above. Eleven of the 15 patients with a recurrence died of tumor-related causes during follow-up whereas none of the non-recurring patients did. None of the patients were treated with radio- or chemo-therapy. Among the 26 patients, five patients did not receive mitotane throughout their follow-up (including Patient 2 with a follow-up of less than 3 years), 18 patients received mitotane as adjuvant therapy (including Patient 11 with a follow-up of less than 3 years) and three patients received mitotane after recurrence as palliative therapy (Table 1). All the treated patients received mitotane (adjuvant or palliative treatment) after blood sampling except Patients 5 and 8 (group NR3yrs) who received mitotane 0.9 and 1.8 months before blood sampling, respectively. Patient 5 reached mitotane therapeutic levels only 8 months later, while in Patient 8, mitotane concentration was only 4.3 mg/L at the time of blood collection.

### 2.2. MiR-483-5p Levels and Recurrence-Risk Within 3 Years: Analysis of the Group R < 3yrs vs. the Group NR3yrs

**Figure 2:**
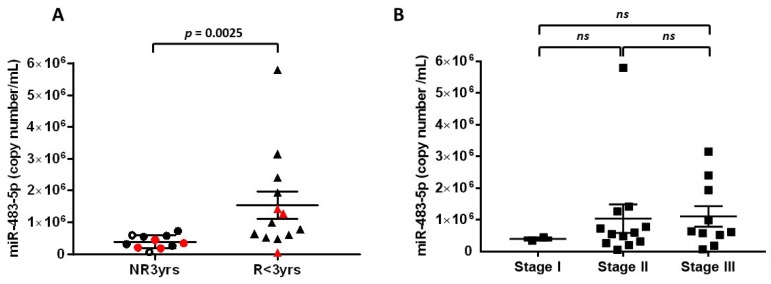
Post-operative serum levels of miR-483-5p in ACC patients. (**A**) Comparison of miR-483-5p levels in NR3yrs (*n* = 11) and R < 3yrs groups (*n* = 13). Cycle threshold (Ct) values were converted to absolute number of copies/mL using a dilution series of a known input quantity of synthetic target miRNA run simultaneously with the experimental samples. Statistically significant difference was assessed using Mann–Whitney test (*p* = 0.0025). Black circles and triangles: patients who received mitotane adjuvant therapy after serum sampling. White circles in the NR3yrs group: Patients 5 and 8 received mitotane adjuvant therapy 0.9 and 1.8 months before serum sampling, respectively. Red symbols: patients with no mitotane therapy throughout their follow-up. (**B**) Comparison of miR-483-5p levels in NR3yrs and R < 3yrs patients in function of ENSAT stage (stage I: *n* = 2; stage II: *n* = 12; stage III: *n* = 10); ns: non-significant. The lines within the scatter plot represent the mean ± S.E.M of miRNA copy number/mL.

**Figure 3:**
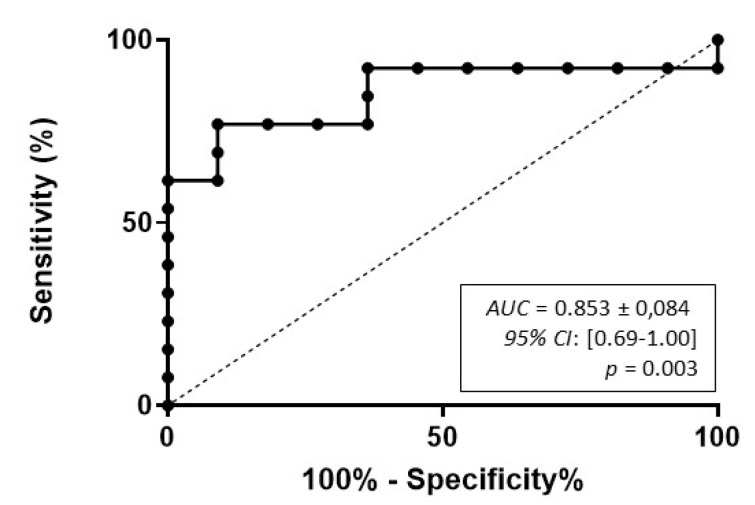
Receiver operating characteristic (ROC) curve analysis for miR-483-5p absolute copy number in ACC patients. Three months-post-operative serum samples can discriminate between NR3yrs and R < 3yrs patients with significant accuracy. The area under the curve (AUC), the 95% confidence interval (CI) and the *p* value are indicated.

To analyze the link between serum miR-483-5p levels and recurrence within 3 years, we compared serum miR-483-5p levels in the group R < 3yrs (*n* = 13) vs. the group NR3yrs (*n* = 11). Figure 2a shows that the mean of miR-483-5p copy number was significantly higher in the group R < 3yrs as compared to the group NR3yrs: 1,541,990 ± 428,377 copies/mL vs. 388,457 ± 62,169 copies/mL, respectively (*p* = 0.0025). As described in detail in the “Patients” section, 16 patients received mitotane as adjuvant therapy after serum sampling, so that for these 16 patients, we excluded any potential pharmacological interaction between mitotane and miR-483 5p levels. Patients 5 and 8 in the NR3yrs group received mitotane 0.9 and 1.8 months before serum sampling. Nevertheless, Patient 5 reached mitotane therapeutic levels only 8 months later, while in Patient 8, mitotane concentration was only 4.3 mg/L, so that for these two patients as well, it seemed unlikely that mitotane could have been involved in a pharmacological interaction with miR-483-5p. Moreover, exclusion of both Patients 5 and 8 from the NR3yrs group from the statistical analysis maintained a significant difference between NR3yrs and R < 3yrs patients (*p* = 0.004. Comparison between ENSAT stage I (*n* = 2), stage II (*n* = 12) and stage III (*n* = 10) ACC patients revealed no significant difference in their respective circulating miR-483-5p levels within 3 months post-surgery (stage I: 395,942 ± 50,997 copies/mL; stage II: 1,036,983 ± 448 289 copies/mL; stage III: 1,108,321 ± 326,412 copies/mL; *p* = 0.474 Kruskal–Wallis multiple comparisons test) (Figure 2b). Receiver operating characteristic (ROC) curve analysis was performed to determine the diagnostic performance of miR-483-5p in discriminating between the R < 3yrs group and the NR3yrs group (Figure 3). High diagnostic accuracy was observed for miR-483-5p with an area under the curve (AUC) of 0.853 ± 0.084 (95% confidence interval (CI): 0.69–1.00, *p* = 0.003). As detailed in the “Statistical analyses” section, we chose a cut-off value for serum miR-483-5p level of 752,898 copies/mL, which predicted recurrence within 3 years with 100% specificity (95% CI: 71.5–100) and 61.5% sensitivity (95% CI: 31.6–86.1). Using this cut-off, no association was found between miR-483-5p levels and ENSAT stage (Fisher’s exact test: *p* = 0.673; low miR-483-5p: <752,898 copies/mL and high miR-483-5p: >752,898 copies/mL).

**Figure 4:**
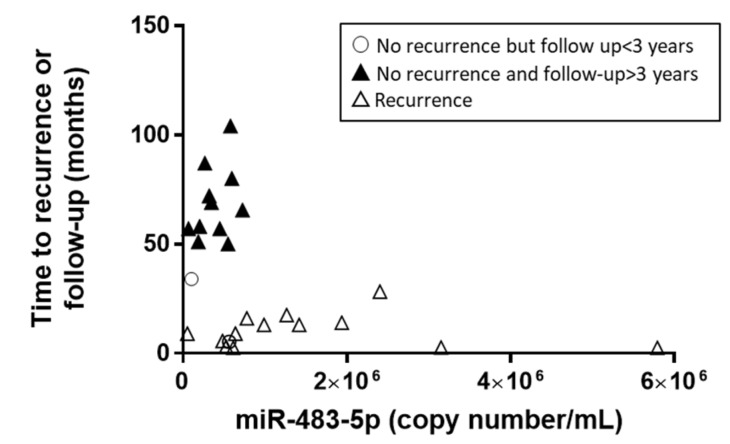
Time to recurrence or follow-up with no recurrence in function of miR-483-5p copy number.

Remarkably, an inverse correlation between miR-483-5p copy numbers within the 3 months post-surgery and time to recurrence or last follow-up with no recurrence was observed, with the identification of two distinct groups of patients (Spearman *r* = −0.42, *p* = 0.03, Figure 4).

### 2.3. Survival Predictive Value of miR-483-5p Levels in 3 Months Post-Operative Serum Samples

**Figure 5:**
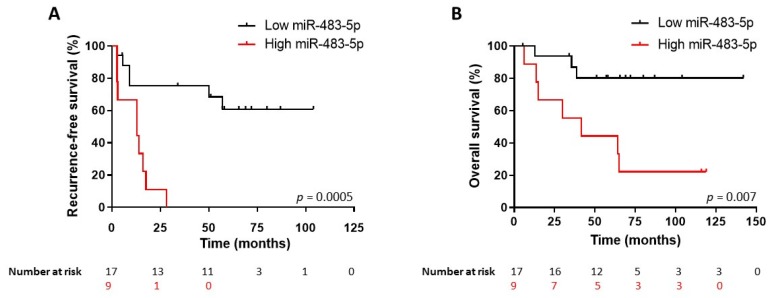
Kaplan–Meier survival analysis of all 26 patients (NR3yrs *n* = 11, R < 3yrs *n* = 13 and two patients with no recurrence and follow-up <3 years) according to miR-483-5p circulating levels. (**A**) Recurrence-free survival (RFS). High levels of miR-483-5p (>752,898 copies/mL cut-off value) within the 3-month post-surgery period predict a shorter RFS (Log-rank *p* = 0.0005). (**B**) Overall survival. High levels of miR-483-5p (>752,898 copies/mL cut-off value) within the 3-month post-surgery period predict a shorter OS (Log-rank *p* = 0.007).

**Figure 6:**
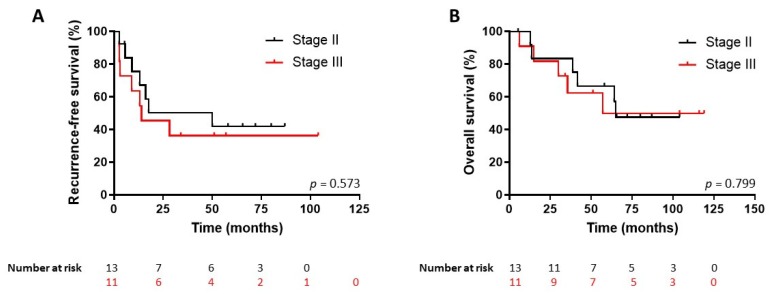
Kaplan–Meier survival analysis according to European Network for the Study of Adrenal Tumors (ENSAT) stage II and stage III (24 patients). No significant differences were observed between ENSAT stage II and stage III for Recurrence-free survival (**A**) or Overall survival (**B**) predictions in this cohort.

We then used the ROC-derived miR-483-5p cut-off value to perform a Kaplan–Meier analysis of recurrence-free (RFS) and overall survival (OS) in the whole cohort of 26 patients, including not only the 13-patient group R < 3yrs and the 11-patient group NR3yrs but also the two patients with no recurrence but a follow-up < 3yrs. Figure 5 shows that patients with a serum miR-483-5p level higher than 752,898 copies/mL had a significant shorter RFS (*n* = 9; median RFS 1.1 years) than the group with miR-483-5p level lower than 752,898 copies/mL (*n* = 17; median RFS >8.5 years) (hazard ratio (HR) for recurrence: 4.80; 95% CI: 1.46–15.78; log-rank test *p* = 0.0005). Likewise, patients with high levels of miR-483-5p had also a poorer OS (*n* = 9; median OS 3.5 years) as compared to the low miR-483-5p group (*n* = 17, median OS >11.8 years) (HR for death: 5.23; 95% CI: 1.36–20; log-rank test *p* = 0.007) (Figure 5). By contrast, ENSAT stage II or III patients showed no significant difference in RFS (Stage II: *n* = 13, median RFS 4.2 years. Stage III: *n* = 11, median RFS 1.2 years) (HR for recurrence: 1.34; 95% CI: 0.46–3.88, log-rank test *p* = 0.57) or OS (Stage II: median OS 5.4 years; Stage III: median OS 4.7 years) (HR for death: 1.16; 95% CI: 0.35–3.86, log-rank test *p* = 0.79)] (Figure 6).

### 2.4. The Prognostic Value of miR-483-5p is Independent from Ki67 Index and Tumor Stage

Univariate analyses for age, sex and clinical parameters (tumor size, Ki67, ENSAT stage) were performed. None of the clinical parameters reached significance for RFS or OS predictions in this cohort (Table 2). While the circulating miR-483-5p level (cut-off 752,898 copies/mL) did not reach significance for OS (HR = 2.79, *p* = 0.09), it was found to be the single significant predictor of disease recurrence with an HR of 2.79 (*p* = 0.007) (Table 2). Upon multivariate analyses involving tumor size, Ki67, ENSAT stage and miR-483-5p level, miR-483-5p retained a strong prognostic power for RFS with an HR = 5.9 (*p* = 0.011) (Table 3). Of note, Ki67 >10% was not significantly associated with OS in univariate and multivariate analyses but a trend for shorter RFS was detectable in both analyses (univariate analysis HR: 0.38, *p* = 0.083; multivariate analysis HR: 0.36; *p* = 0.092).

## 3. Discussion

Despite significant advances in the characterization of the ACC molecular landscape and the identification of distinct molecular subtypes with different outcomes, major efforts are still needed to improve diagnosis, surveillance and treatment of patients with ACC. In the last five years, circulating miRNAs have emerged as promising non-invasive biomarkers for adrenocortical cancer. Among candidate miRNAs, miR-483-5p was found to be increased in pre-operative serum or plasma samples from ACC patients [5,12,13,14]. MiR-483 gene, which is located at 11p15.5 within the second intron of IGF2 gene, is overexpressed in ACC in correlation with the well documented IGF2 overexpression in this type of tumors [9], suggesting the tumor origin of circulating miR-483-5p. We have previously shown that miR-483-5p levels in pre-operative serum are predictive of recurrence risk in ACC [5]. Interestingly, extracellular vesicle-associated miR-483-5p was reported to display a high accuracy in the pre-operative diagnosis of ACC [15]. Significant differences in miR-483-5p levels were found between ENSAT stage I/II and stage III/IV in pre- and post-surgery plasma samples [14]. However, in this work, time interval between surgery and serum sampling was not clearly stated and the potential impact of mitotane therapy on circulating miR-483-5p levels was not evaluated. Indeed, while 40 % of the samples were collected in the 6 months after surgery, no information was given for the remaining [14]. Hence, heterogeneous post-operative sampling may have introduced imprecisions in the analysis due to complex dynamic changes of miRNA in the circulation in the long term. Furthermore, 44 samples were analyzed in 27 patients indicating that some patients were arbitrarily weighted more than others. In the present work, we measured circulating miR-483-5p absolute levels in ACC patients based on three criteria: (1) tumors with or without recurrence within 3 years post-surgery, (2) serum sampling within 3 months post-surgery and (3) one sample per patient (the first one). We show that high circulating miR-483-5p within 3 months post-surgery was associated with more than a four-fold increased risk of recurrence and was predictive of poor overall survival for ACC patients. In multivariate analysis, miR-483-5p appeared as a significant prognostic factor, independent of the well-established prognostic factors, including ENSAT stage and Ki67.

Methodologically, we used the sensitive and validated Taqman PCR assays and the exogenous spiked-in cel-miR-39 with standard curves. We derived a cut-off value of miR-483-5p copy numbers from ROC curve, which distinguishes between recurring and non-recurring ACC patients with a high diagnostic accuracy (AUC of 0.853). These results suggest that miR-483-5p is a good candidate miRNA to monitor disease evolution. In the same line, we have previously reported a correlation between tumor size and circulating miR-483-5p levels, which were decreased after surgery [5]. Importantly, our miR-483-5p measurements were performed in sera sampled before mitotane adjuvant or palliative therapy, thus excluding a potential effect of mitotane on circulating miR-483-5p concentrations. For the only two patients who received mitotane about 1-2 months before serum sampling, it seemed unlikely that mitotane could have an impact on miR-483-5p levels for the reasons outlined previously (Results section). Serum miR-483-5p-based prediction of responsiveness to mitotane in ACC deserves future investigations in prospective longitudinal studies. We also addressed the potential confounding effect of post-surgical mitotane treatment on the risk of recurrence. As this treatment was shown to have a positive impact on recurrence risk, we determined whether the difference that we measured in recurrence rate between patients with high vs. low levels of circulating miR-483-5p is free from any bias linked to different exposure to post-surgical mitotane therapy between the NR3yrs and R < 3yrs groups of patients. In the group of 17 patients who all had mitotane adjuvant therapy, a significant difference in miR-483-5p levels was maintained between patients who recurred before 3 years (*n* = 10) vs. patients who did not recur before 3 years (*n* = 7), (*p* = 0.0136). This indicates that miR-483-5p is still a prognostic factor even in patients who received post-surgical mitotane therapy.

The main limitations of our study are the small size of the cohort and a single-center population. While the prognostic values of the ENSAT stage and Ki67 index have been largely validated for ACC management [16], we found here in this cohort that ENSAT stage II and III or Ki67 > 10% did not predict recurrence risk or death. This is likely due to the limited number of patients analyzed in this study. However, with the same number of patients, the concentration of circulating miR-483-5p is able to distinguish between patients with different prognoses of recurrence and death. We found that miR-483-5p detection in post-operative samples 3 months post-surgery was not significantly different between tumor ENSAT stage II (*n* = 13) and stage III (*n* = 11) patients. This is in contrast to the results reported by Salvianti et al. [14] showing that post-operative miR-483-5p levels were significantly different between stage I/II (*n* = 14) and III/IV (*n* = 9) groups. However, our study design is different as we excluded metastatic patients at diagnosis (stage IV ACC) and narrowed sampling time to 3 months post-surgery. Accordingly, we demonstrate the strong prognostic power of circulating post-operative miR-483-5p for recurrence in univariate and multivariate analyses.

At the cellular level, we have recently shown that miR-483-5p promotes ACC aggressiveness by targeting the N-myc-Downstream Regulated Gene member 2 (NDRG2) in NCI H295R cell line [17]. In another work, inhibition of miR-483-5p in NCI-H295R cells was reported to also reduce their proliferation [8]. Recent studies have shown that tumors can actively release microvesicle-containing miRNAs into the peripheral circulation to exchange information with distant cells and promote metastatic spread [18]. Whether extra-cellular circulating miR-483-5p is involved in ACC progression and dissemination deserves future investigations.

## 4. Materials and Methods

### 4.1. Patients and Clinical Samples

We conducted a retrospective, single-center study, based on a collection of post-operative sera sampled in patients followed for ACC at the Endocrinology and Diabetes Unit of the University Hospital of Würzburg, and who were not metastatic at diagnosis (ENSAT stages I, II, and III) and operated between the years 2002 and 2013. All patients were registered in the ENSAT database. The study was approved by the ethics committee of the University of Würzburg (Germany) (#88/11), and written informed consent was obtained from all subjects. Two hundred and four post-operative sera from 48 patients were available. However, sample collection time points after surgery were highly heterogeneous. To evaluate the prognostic value of miR-483-5p more accurately, we selected 26 patients with at least one serum sampled within 3 months post-operatively. For each patient, a single serum sample was analyzed for the presence of miR-483-5p, which was the first serum sampled within 3 months after surgery (sampling interval ranging from 13 to 90 days).

### 4.2. RNA Extraction

Serum samples were thawed on ice and centrifuged at 3 000 g for 5 min. Total RNA was extracted from 450 µL of supernatant using the miRVana PARIS kit (ThermoFisher Scientific, Courtaboeuf, France) according to the manufacturer’s instructions. Synthetic non-human miRNA (C. elegans cel-miR-39, 25 fmol) was added to each denatured sample prior to extraction to adjust for differences in RNA recovery.

### 4.3. Quantification of Serum miRNA by Reverse Transcription-Quantitative PCR (RT-qPCR)

MicroRNAs were quantified by RT-qPCR as described previously in details [5]. Following RNA extraction, reverse transcription was performed using Taqman miRNA Reverse Transcription Kit and miRNA-specific stem-loop primers (ThermoFisher Scientific). Briefly, a fixed volume of 2.5 µL from the 45 µL RNA eluate was used as input into the RT reaction. In parallel, a 7-point standard curve ranging from 3 × 10^8^ to 3 × 10^2^ copies was reverse-transcribed for each miRNA using synthetic miRNA mimics (ThermoFisher Scientific). Real-time PCR was performed in duplicate using 4.5 µL of the 5-fold diluted RT product combined with 5.5 µL of PCR assay reagents (Taqman miRNA assays, ThermoFisher Scientific) to generate a final PCR volume of 10 µL (Assay ID: hsa-miR-483-5p: 002338; Cel-miR-39: 000200). Assays were run on C1000 Thermal cycler (CFX96 Real Time system, Bio-Rad, Marnes-La-Coquette, France) at 95 °C for 10 min, followed by 40 cycles of 95 °C for 15 s and 60 °C for 1 min. Data were analyzed with CFX Manager Software version 3.1 (Bio-Rad). Copy number of miR-483-5p in each sample was normalized to C. elegans exogenous spike-in control (Cel-miR-39) using a previously published median normalization procedure, and expressed as normalized copy number per mL of serum [5].

### 4.4. Statistical Analyses

Statistical analyses were performed using GraphPad Prism software version 7.04 (San Diego, CA, USA). Mann–Whitney test was performed to compare miRNA expression levels, based on the results of D’Agostino and Pearson normality test. Results are expressed as means ± standard error of the mean (S.E.M). Receiver operating characteristic (ROC) curve was generated to determine miR-483-5p diagnostic value. Calculation of the Youden index yielded an optimal cut-off of miR-483-5p > 603,601 copies/mL (sensitivity 76.9 % and specificity 90.9 %). However, we chose to raise the cut-off to 752,898 copies/mL to obtain a maximal specificity (100% specificity and 61.5% sensitivity). High expression corresponds to miR-483-5p levels > 752,898 copies/mL while low expression corresponds to miR-483-5p levels < 752,898 copies/mL. Univariable and multivariable Cox regression models were used to determine the significance of the clinical characteristics for prognosis of RFS and OS. Univariate analyses were performed for quantitative variables (age, tumor size), qualitative variables (sex, ENSAT stage) and according to cut-off values (Ki67 > 10% and miR-483-5p >752,898 copies/mL). Multivariate hazard ratios (HRs) were determined for tumor size, Ki67, ENSAT stage and miR-483-5p. Survival analyses were performed using the Kaplan–Meier method and curves were compared using the log-rank test. For all the analyses, a *p* < 0.05 value was considered as statistically significant.

## 5. Conclusions

The role of miRNA in the physiopathology of ACC remains poorly understood. Nonetheless, miR-483-5p was consistently found to be upregulated in the tumor as well as in the serum/plasma of ACC patients, thus positioning this miRNA among the hallmarks of adrenocortical cancer. Our study goes further to demonstrate that early detection of miR-483-5p post-operatively has a powerful prognostic value. Indeed, high miR-483-5p levels in sera sampled within 3 months post-surgery were significantly associated with reduced overall survival and recurrence-free survival. Circulating miR-483-5p thus appears as a potent non-invasive biomarker for therapeutic decision-making during ACC patient surveillance and deserves further validation in prospective clinical studies.

## Figures and Tables

**Table 1:** Clinical characteristics of the 26 patients with adrenocortical cancer (ACC) with serum sampled within 3 months after surgery.

Patient Number	Age	Sex	Ensat Stage	Mitotane Post-op Adjuvant Therapy	Recur-rence	Time to Recurrence (Months)	Death	Time to Death (Months)	Last Follow-up Without Recurrence or Death (Months)	Group
**1**	38	F	I	N	N	-	N	-	69	NR3yrs
**2**	30	F	II	N	N	-	N	-	5.3	-
**3**	60	M	II	N	N	-	N	-	58	NR3yrs
**4**	52	F	II	Y	N	-	N	-	72	NR3yrs
**5**	70	F	II	Y ^a^	N	-	N	-	80	NR3yrs
**6**	46	F	II	Y	N	-	N	-	87	NR3yrs
**7**	21	M	II	Y	N	-	N	-	65.5	NR3yrs
**8**	48	F	III	Y ^a^	N	-	N	-	57	NR3yrs
**9**	64	M	III	N	N	-	N	-	51	NR3yrs
**10**	70	F	III	Y	N	-	N	-	104	NR3yrs
**11**	53	M	III	Y	N	-	N	-	34	-
**12**	44	F	I	N	Y	57	N	-	142	NR3yrs
**13**	42	F	II	Y	Y	50	N	-	104	NR3yrs
**14**	59	F	II	Y	Y	5.65	Y	12.7	-	R < 3yrs
**15**	56	M	II	Y	Y	2.6	Y	13.5	-	R < 3yrs
**16**	40	F	II	N ^b^	Y	9.1	Y	38.7	-	R < 3yrs
**17**	66	F	II	N ^b^	Y	17.6	Y	41.6	-	R < 3yrs
**18**	46	F	II	N ^b^	Y	13	Y	64	-	R < 3yrs
**19**	42	M	II	Y	Y	16	Y	65	-	R < 3yrs
**20**	62	M	III	Y	Y	9	Y	57	-	R < 3yrs
**21**	51	F	III	Y	Y	3	Y	6	-	R < 3yrs
**22**	78	F	III	Y	Y	2.7	Y	14.9	-	R < 3yrs
**23**	37	M	III	Y	Y	13	Y	30	-	R < 3yrs
**24**	47	F	III	Y	Y	2.6	Y	35.5	-	R < 3yrs
**25**	47	M	III	Y	Y	14	N	-	119	R < 3yrs
**26**	43	F	III	Y	Y	28.3	N	-	116	R < 3yrs

NR3yrs: patient with no recurrence after 3 years of post-surgical follow-up. R < 3yrs: patient with a recurrence before 3 years of follow-up. Time to recurrence and time to death were calculated from the date of surgery. F: female; M: male; N: no; Y: yes. ^a^, Among the patients treated post-operatively with mitotane adjuvant therapy, Patients 5 and 8 received mitotane 0.9 and 1.8 months before serum sampling, respectively. All other patients received mitotane after serum sampling including patients 16, 17 and 18 who received palliative mitotane treatment ^b^.

**Table 2:** Univariate analysis (Cox regression) of circulating miR-483-5p and clinicopathological parameters in relation to overall survival (OS) and recurrence-free survival (RFS).

Variable	OS			RFS		
	HR	95% CI	*p* Value	HR	95% CI	*p* Value
Age	1.02	0.97–1.07	0.373	1.01	0.97–1.05	0.601
Sex	0.90	0.26–3.08	0.862	0.87	0.28–2.68	0.817
Tumor size	1.02	0.92–1.13	0.684	1.05	0.96–1.15	0.274
Ki67 > 10%	0.45	0.12–1.69	0.239	0.38	0.11–1.26	0.083
ENSAT stage I–II vs. III	0.70	0.21–2.33	0.566	0.54	0.18–1.61	0.269
miR-483-5p cut-off	2.79	0.85–9.19	0.090	4.84	1.53–15.3	**0.007**

Univariate analysis includes quantitative variables (age; tumor size), qualitative variables (sex; ENSAT stage I, II vs. III), and dichotomized variables (Ki67 > 10% as the reference category; miR-483-5p < 752,898 copy number/mL as the reference category). HR: hazard ratio; 95% CI: 95% confidence interval. Bold numbers indicate a significant difference.

**Table 3:** Multivariate analysis (Cox regression) of circulating miR-483-5p and clinicopathological parameters in relation to overall survival (OS) and recurrence-free survival (RFS).

Variable	OS			RFS		
	HR	95% CI	*p* Value	HR	95% CI	*p* Value
Tumor size	0.92	0.80–1.05	0.212	0.90	0.79–1.03	0.125
Ki67 > 10%	0.44	0.11–1.75	0.245	0.36	0.10–1.33	0.092
ENSAT stage I–II vs. III	0.41	0.09–1.92	0.260	0.28	0.06–1.32	0.109
miR-483-5p cut-off	3.11	0.66–14.6	0.150	5.98	1.30–27.6	**0.011**

Multivariate analysis includes tumor size (continuous variable), Ki67 (dichotomized variable with Ki67 > 10% as the reference category), ENSAT stage (I, II vs. III) and miR-483-5p (dichotomized variable with miR-483-5p < 752,898 copy number/mL as the reference category). HR: hazard ratio; 95% CI: 95% confidence interval. Bold number indicate a significant difference.

## References

[1] Fassnacht M., Johanssen S., Quinkler M., Bucsky P., Willenberg H.S., Beuschlein F., Terzolo M., Mueller H.H., Hahner S., Allolio B. (2009). Limited prognostic value of the 2004 International Union Against Cancer staging classification for adrenocortical carcinoma: Proposal for a Revised TNM Classification. Cancer.

[2] Else T., Kim A.C., Sabolch A., Raymond V.M., Kandathil A., Caoili E.M., Jolly S., Miller B.S., Giordano T.J., Hammer G.D. (2014). Adrenocortical carcinoma. Endocr. Rev..

[3] Jouinot A., Bertherat J. (2018). Management of endocrine disease: Adrenocortical carcinoma: Differentiating the good from the poor prognosis tumors. Eur. J. Endocrinol..

[4] Lughezzani G., Sun M., Perrotte P., Jeldres C., Alasker A., Isbarn H., Budaus L., Shariat S.F., Guazzoni G., Montorsi F. (2010). The European Network for the Study of Adrenal Tumors staging system is prognostically superior to the international union against cancer-staging system: A North American validation. Eur. J. Cancer.

[5] Chabre O., Libe R., Assie G., Barreau O., Bertherat J., Bertagna X., Feige J.J., Cherradi N. (2013). Serum miR-483-5p and miR-195 are predictive of recurrence risk in adrenocortical cancer patients. Endocr. Relat. Cancer.

[6] Ambros V. (2004). The functions of animal microRNAs. Nature.

[7] Cherradi N. (2015). microRNAs as Potential Biomarkers in Adrenocortical Cancer: Progress and Challenges. Front. Endocrinol. (Lausanne).

[8] Ozata D.M., Caramuta S., Velazquez-Fernandez D., Akcakaya P., Xie H., Hoog A., Zedenius J., Backdahl M., Larsson C., Lui W.O. (2011). The role of microRNA deregulation in the pathogenesis of adrenocortical carcinoma. Endocr. Relat. Cancer.

[9] Patterson E.E., Holloway A.K., Weng J., Fojo T., Kebebew E. (2011). MicroRNA profiling of adrenocortical tumors reveals miR-483 as a marker of malignancy. Cancer.

[10] Soon P.S., Tacon L.J., Gill A.J., Bambach C.P., Sywak M.S., Campbell P.R., Yeh M.W., Wong S.G., Clifton-Bligh R.J., Robinson B.G. (2009). miR-195 and miR-483-5p Identified as Predictors of Poor Prognosis in Adrenocortical Cancer. Clin. Cancer Res..

[11] Assie G., Letouze E., Fassnacht M., Jouinot A., Luscap W., Barreau O., Omeiri H., Rodriguez S., Perlemoine K., Rene-Corail F. (2014). Integrated genomic characterization of adrenocortical carcinoma. Nat. Genet..

[12] Patel D., Boufraqech M., Jain M., Zhang L., He M., Gesuwan K., Gulati N., Nilubol N., Fojo T., Kebebew E. (2013). MiR-34a and miR-483-5p are candidate serum biomarkers for adrenocortical tumors. Surgery.

[13] Szabo D.R., Luconi M., Szabo P.M., Toth M., Szucs N., Horanyi J., Nagy Z., Mannelli M., Patocs A., Racz K. (2014). Analysis of circulating microRNAs in adrenocortical tumors. Lab. Investig..

[14] Salvianti F., Canu L., Poli G., Armignacco R., Scatena C., Cantini G., Di Franco A., Gelmini S., Ercolino T., Pazzagli M. (2017). New insights in the clinical and translational relevance of miR483-5p in adrenocortical cancer. Oncotarget.

[15] Perge P., Butz H., Pezzani R., Bancos I., Nagy Z., Paloczi K., Nyiro G., Decmann A., Pap E., Luconi M. (2017). Evaluation and diagnostic potential of circulating extracellular vesicle-associated microRNAs in adrenocortical tumors. Sci. Rep..

[16] Fassnacht M., Dekkers O.M., Else T., Baudin E., Berruti A., de Krijger R., Haak H.R., Mihai R., Assie G., Terzolo M. (2018). European Society of Endocrinology Clinical Practice Guidelines on the management of adrenocortical carcinoma in adults, in collaboration with the European Network for the Study of Adrenal Tumors. Eur. J. Endocrinol..

[17] Agosta C., Laugier J., Guyon L., Denis J., Bertherat J., Libe R., Boisson B., Sturm N., Feige J.J., Chabre O. (2018). MiR-483-5p and miR-139-5p promote aggressiveness by targeting N-myc downstream-regulated gene family members in adrenocortical cancer. Int. J. Cancer.

[18] Tkach M., Thery C. (2016). Communication by Extracellular Vesicles: Where We Are and Where We Need to Go. Cell.

